# Lipid Metabolism Regulates Oxidative Stress and Ferroptosis in RAS-Driven Cancers: A Perspective on Cancer Progression and Therapy

**DOI:** 10.3389/fmolb.2021.706650

**Published:** 2021-08-16

**Authors:** Caterina Bartolacci, Cristina Andreani, Yasmin El-Gammal, Pier Paolo Scaglioni

**Affiliations:** Department of Internal Medicine, University of Cincinnati College of Medicine, Cincinnati, OH, United States

**Keywords:** lipid metabolism, ferroptosis, tumorigenesis, oxidative stress, RAS oncogenes

## Abstract

*HRAS*, *NRAS* and *KRAS*, collectively referred to as oncogenic RAS, are the most frequently mutated driver proto-oncogenes in cancer. Oncogenic RAS aberrantly rewires metabolic pathways promoting the generation of intracellular reactive oxygen species (ROS). In particular, lipids have gained increasing attention serving critical biological roles as building blocks for cellular membranes, moieties for post-translational protein modifications, signaling molecules and substrates for ß-oxidation. However, thus far, the understanding of lipid metabolism in cancer has been hampered by the lack of sensitive analytical platforms able to identify and quantify such complex molecules and to assess their metabolic flux *in vitro* and, even more so, in primary tumors. Similarly, the role of ROS in RAS-driven cancer cells has remained elusive. On the one hand, ROS are beneficial to the development and progression of precancerous lesions, by upregulating survival and growth factor signaling, on the other, they promote accumulation of oxidative by-products that decrease the threshold of cancer cells to undergo ferroptosis. Here, we overview the recent advances in the study of the relation between RAS and lipid metabolism, in the context of different cancer types. In particular, we will focus our attention on how lipids and oxidative stress can either promote or sensitize to ferroptosis RAS driven cancers. Finally, we will explore whether this fine balance could be modulated for therapeutic gain.

## Clinical Significance of RAS Mutations

The three *RAS* genes (*HRAS*, *NRAS* and *KRAS*), hereafter collectively referred to as oncogenic RAS, are the most frequently mutated driver proto-oncogenes in cancer, with *KRAS* being the most prevalent. Notably, mutant KRAS is present in more than 90% of pancreatic ductal adenocarcinoma (PDAC) where it is the most frequent and earliest genetic alteration, as it is found in more than 90% of neoplastic precursor lesions (*e.g*. pancreatic intraepithelial neoplasia, PanINs) (Kanda et al., 2012; Eser et al., 2014). Similarly, mutant KRAS is present in 30–40% of colorectal cancers (CRC) and almost 25% of patients with Non-Small Cell Lung Cancer (NSCLC), where it correlates with poor prognosis and high risk of recurrence (Stephen et al., 2014).

While much of the early work had focused on the signal transduction related to cell proliferation, it is now understood the RAS oncogene has yet other crucial roles in tumorigenesis. For instance, it orchestrates the reprogramming of lipid metabolism and promotes the generation of intracellular reactive oxygen species (ROS). Since these metabolic changes are critical for ferroptosis, a unique form of iron-dependent programed cell death, and are dependent on the presence of oncogenic RAS, they might offer new therapeutic opportunities.

## An Introduction to Ferroptosis and Lipid Peroxidation

Ferroptosis (extensively reviewed in (Dixon and Stockwell, 2019; Zheng and Conrad, 2020) is a unique form of iron-dependent programed cell death defined by the existence of substantial oxidative stress and lipid peroxidation (LPO). It differs from other well-characterized types of cell death as apoptosis, pyroptosis, necroptosis or autophagy in morphology, biochemistry, and genetics. Accordingly, inhibitors for apoptosis, necrosis or autophagy are all ineffective against ferroptosis (Dixon et al., 2012).

Even if preliminary observations were reported as early as in the 70s (Maellaro et al., 1990), only in 2012 the term “ferroptosis” was first introduced by the group of Dr. Stockwell (Dixon et al., 2012) to finally provide a rational explanation for the long-lasting query regarding the nature of LPO-induced cell death.

LPO was first studied in relation to damage to alimentary oils and fats in meat and meat products (Dianzani and Barrera, 2008), but was soon implicated in numerous pathological states, including cancer. It can be generally described as a complex process whereby oxidants, free radicals or nonradical species, attack lipids containing carbon-carbon double bond(s), resulting in the formation/propagation of lipid hydroperoxides (LOOH) and peroxyl radicals, which in turn generate secondary products with prolonged half-life.

Thus, understanding LPO entails a detailed knowledge of lipids and oxidative stress, which we will briefly address with particular attention to their relationship with oncogenic RAS.

It is now well-established that LPO plays a central role in the initiation and execution of ferroptosis and that LPO-induced toxic species, such as lipid derived toxic aldehydes, are biomarkers of ferroptosis. However, the identification of the lipid species that are essential for the regulation, initiation and execution of ferroptosis remain poorly understood. Even more so, analyzing ferroptosis *in vivo* remains challenging. Indeed, exploring ferroptosis requires lipidomic and redox analyses that are technically demanding, giving the huge diversity and biochemical complexity of lipids. In addition, none of the biomarkers or gene products identified to date is entirely specific to ferroptosis. The unambiguous demonstration of the occurrence of ferroptosis requires the simultaneous detection of biochemical markers of LPO, redox-active iron, and deficiency in the repair of the lipid peroxides (Dixon and Stockwell, 2019).

Today, ferroptosis is the subject of intense investigation and its clinical relevance has started to being recognized. Indeed, various compounds, some of which are FDA-approved drugs, have been identified as ferroptosis inducers in cancer cells (Shen et al., 2018; Hassannia et al., 2019).

Ferroptosis was initially found to be induced by a set of small molecules identified in a screen for compounds able to selectively induce cell death in isogenic cancer cell lines tumors carrying a mutant form of RAS, suggesting a connection between RAS oncogene and ferroptosis (Dolma et al., 2003; Yagoda et al., 2007; Yang and Stockwell, 2008). However, subsequent studies have questioned the selective lethality of these compounds on RAS-mutated cell lines (Yang and Stockwell, 2008). Moreover, while cancer cells display high levels of oxidative stress, increased levels of LPO products are detected only in some cancer types, depending on the lipid composition of cellular membranes, presence of inflammation and the level of enzymes able to metabolize LPO products (Canuto et al., 1993; Hammer et al., 1997). Thus, the relationship among cancer, RAS-driven cancers in particular, LPO and ferroptosis still remains controversial.

Here, we will briefly review the mechanisms of oxidative stress, lipid metabolism and LPO and the current understanding of how RAS oncogene regulates these processes to escape ferroptosis, highlighting questions still open for future studies.

## Lipid Metabolism: A Broad Picture

Fatty acids (FA) serve essential roles in cancer cells as they provide constituents for cellular membranes and substrates for energy metabolism to meet the demand for high-rate proliferation. Moreover, FA come in many different flavors, and specific FA are essential to support tumorigenesis and cancer progression.

**FIGURE 1:**
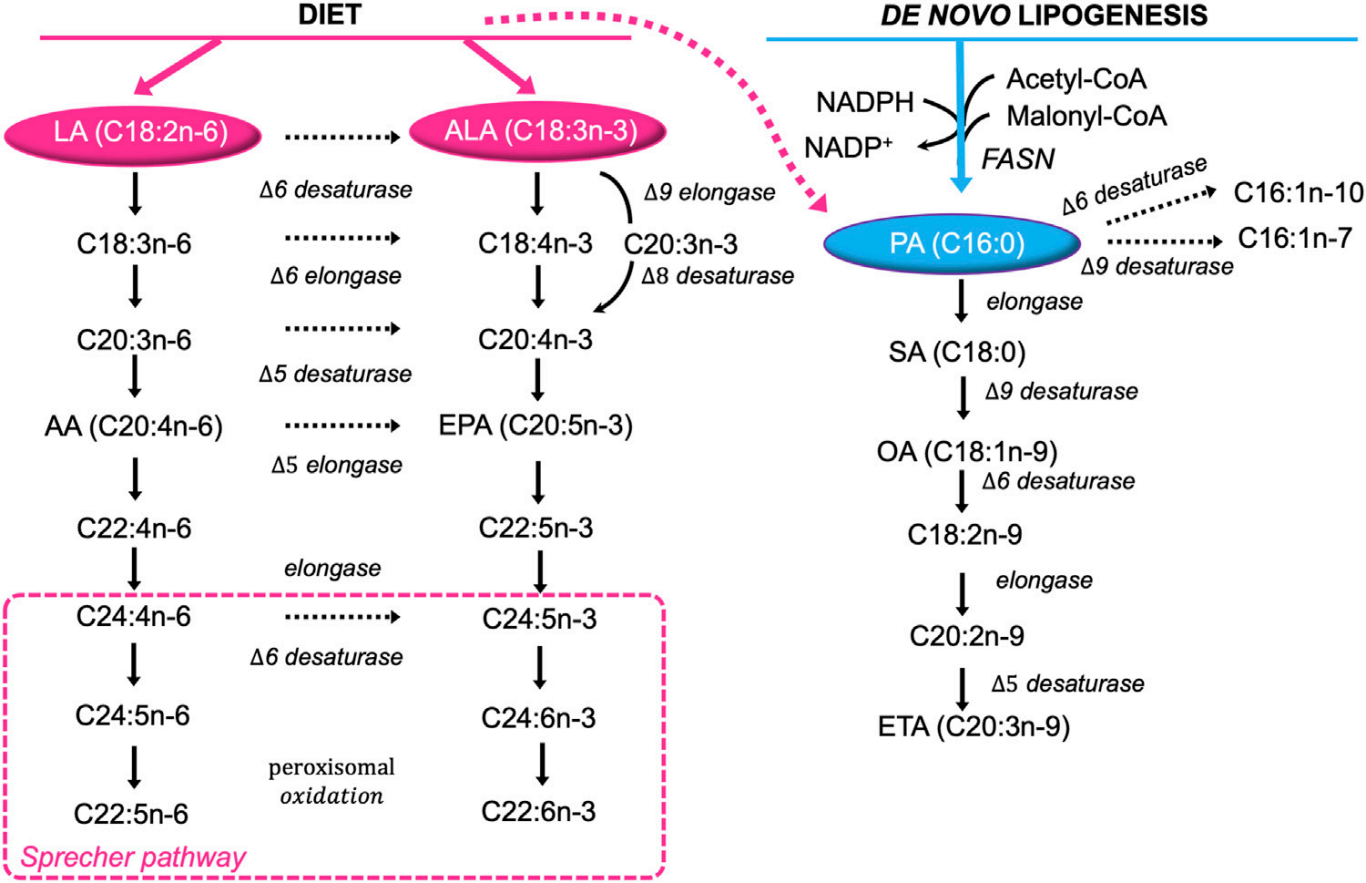
Major pathways of FA desaturation and chain elongation in eukaryotic cells. Both exogenous (diet, pink) and endogenously synthetized FA (blue) are extensively desaturated and elongated giving rise to the huge diversity found in lipid molecules. Note the alternating sequence of desaturation in the horizontal direction and chain elongation in the vertical direction in the formation of polyunsaturated fatty acids from dietary essential fatty acids. LA, linoleic acid; ALA, α-linolenic acid; C18:4n-3, stearidonic acid; C18:3n-6, γ-linolenic acid; C20:3n-3, eicosatrienoic acid; C20:3n-6, dihomo-γ-linolenic acid; C20:4n-3, eicosatetraenoic acid; AA, arachidonic acid; EPA, eicosapentaenoic acid; C22:4n-6, adrenic acid; C22:5n-3, docosapentaenoic acid; C24:4n-6, tetracosatetraenoic acid; C24:5n-6, etracosapentaenoic acid; C24:5n-3, tetracosapentaenoic acid; C24:6n-3, tetracosahexaenoic acid; C22:5n-6, docosapentanoic acid; C22:6n-3, docosahexaenoic acid; PA, palmitic acid; C16:1n-10, sapienic acid; C16:1n-7, palmitoleic acid; OA, oleic acid; SA, stearic acid; C18:2n-9, trans linoleic acid; C20:2n-9, 5,11-eicosadienoic acid; ETA, cis-5,8,11-eicosatrienoic acid.

It is well known that the biosynthesis of saturated FA (SFA) and monounsaturated FA (MUFA) starts from palmitate (PA, C16:0), formed by the 250–270 kDa multifunctional, homodimeric fatty acid synthase (FASN) (Chirala and Wakil, 2004; Asturias et al., 2005; Maier et al., 2006). FASN synthesizes long-chain FA, mainly PA, using acetyl-CoA as a primer, malonyl-CoA as a two-carbon donor, and NADPH as a reducing equivalent. PA is further elongated to stearic acid (SA, C18:0) and/or desaturated to palmitoleic (C16:1n-9) and oleic (OA, C18:1n-9) acids, with the latter being further elongated to eicosatrienoic acid (EA, C20:3n-9) (Miyazaki and Ntambi, 2008) (Figure 1).

However, Δ-6 desaturase shows strong preference for the two essential polyunsaturated fatty acids (PUFA) linoleic acid (LA, C18∶2n-6) and α-linolenic acid (LA, C18∶3n-3) over OA (Sprecher et al., 1995). Hence, eukaryotic cells rely on dietary LA and ALA to synthetize n-6 long chain PUFA (*e.g*. arachidonic acid, AA, C20∶4n-6), and n-3 long chain-PUFA (*e.g*. eicosapentaenoic and docosahexaenoic acids, EPA, C20∶5n-3, DHA, C22∶6n-3), respectively through the “Sprecher pathway” (Voss et al., 1991; Sprecher et al., 1995) (Figure 1).

FA, either *de novo* synthetized or deriving from exogenous sources (i.e. diet), can be broken down into acetyl-CoA, which then enters the tricarboxylic acid (TCA) cycle to aid ATP generation. Alternatively, FA can be incorporated into more complex lipids such as triglycerides (TAG), phospholipids (PL) or cholesteryl esters (CE). Yet, these two distinct pathways require a common initial step known as FA activation by acyl-CoA synthetase (ACS) enzymes (Ellis et al., 2010). FASN is very active during embryogenesis and in fetal lungs, where FA are used for the production of lung surfactant (Wagle et al., 1999). However, in well-nourished adults FASN is less active, as non-transformed cells generally rely on the uptake of lipids from the circulation. By contrast, cancer cells aberrantly activate *de novo* lipid synthesis: in 1953 Medes *et al.* already used *in vivo* labelling with ^14^C-glucose tracer to demonstrate that most of the esterified FA in tumor models were derived from *de novo* synthesis (Medes et al., 1953). The mechanisms underlying the switch of cancer cells to *de novo* lipogenesis remain an area of intense research. (Menendez and Lupu, 2007; Padanad et al., 2016; Rozeveld et al., 2020; Ferraro et al., 2021).

## Oncogenic RAS and Lipid Metabolism: A Fat Addiction

According to the literature, the relationship between oncogenic RAS and lipids is intertwined and multifaceted. Firstly, all the RAS proteins (HRAS, NRAS, KRAS4A, and KRAS4B) are modified by lipids through lipidation which reversibly regulates their membrane localization and function. A RAS plasma membrane anchor consists of two components: a C-terminal S-farnesyl cysteine carboxylmethyl ester, common to all isoforms; and a second signal that comprises mono-palmitoylation of NRAS, duo-palmitoylation of HRAS and a polybasic domain (PBD) of six contiguous lysines in KRAS4B, the predominantly expressed splice variant of KRAS, hereafter referred to as KRAS. Evidence from the Hancock laboratory showed that the anchor of mutant KRAS^G12V^ exhibits remarkable specificity for distinct subclasses of phosphatidylserine (PS). In particular, only in presence of monounsaturated PS, KRAS^G12V^ is assembled into membrane nanoclusters, that are considered to be the hotspots of KRAS activation. On the other hand, KRAS^G12V^ does not interact with fully saturated PS at all, whereas mono- and di-unsaturated PS can support KRAS^G12V^ binding to the plasma membrane, but cannot be assembled into nanoclusters (Zhou et al., 2017). Moreover, full-length KRAS, or its minimal membrane anchor, localizes preferentially to cholesterol-depleted liquid-disordered domains in synthetic model bilayers and KRAS^G12V^ is typically excluded from cholesterol-rich domains, as these domains are suboptimal for Raf activation (Prior et al., 2001; Inder et al., 2008). In agreement, nanoclustering of KRAS (either GDP or GTP-loaded) is insensitive to acute cholesterol depletion (Prior et al., 2003).

The fact that lipid availability and lipid composition of the membrane can deeply impact KRAS localization and function is just the tip of the iceberg. Besides acting as building blocks for membrane assembly, signaling molecules and energy storage, FA have recently been found to serve a pivotal role in coping with oncogenic stress. Our lab and others described that mutant KRAS activation/extinction in preclinical lung cancer (LC) models directly controls the expression of genes involved in β-oxidation and *de novo* lipogenesis, and that this can be exploited for therapeutic gain (Padanad et al., 2016; Gouw et al., 2017; Bartolacci et al., 2021). The role of mutant KRAS in FA oxidation has been reported in a transgenic mouse model that expresses the doxycycline (doxy)-inducible KRAS transgene (KRAS^G12D^) in the respiratory epithelium (Padanad et al., 2016). These mice, when fed with doxy, develop lung tumors that completely regress when doxy is removed with concomitant significant decrease in the expression of lipid metabolism genes (Padanad et al., 2016). In this regard, Acyl-coenzyme A synthetase long chain family member 3 and 4 (*Acsl3* and *Acsl4*) are significantly down regulated in tumors undergoing KRAS^G12D^ extinction and ACSL3 contributes the most to the oncogenic phenotype both *in vitro* and *in vivo*. ACSL enzymes conjugate long-chain FA (12–20 C atoms) with Coenzyme A (CoA) to produce acyl-CoA. While genetic deletion of *Acsl3* in mice does not cause any morphological defects neither during development nor in adult life, it impairs KRAS-driven tumorigenesis (Padanad et al., 2016). Therefore, it may represent a good therapeutic target. Even though a specific inhibitor of ACSL3 is not available, yet, evidence indicates that inhibition of FASN has effects similar to *ACSL3* silencing, opening to new possible therapeutic strategies in NSCLC (Bartolacci et al., 2017, 2021). The role of KRAS in inducing lipogenesis is highlighted by the upregulation of FASN along with other enzymes that control FA metabolism, such as ATP citrate lyase (ACLY) and acetyl-coenzyme A carboxylase (ACC) in the KRAS^G12D^ LC model. Overexpression of both *ACLY* and *FASN* correlates with poor survival and with increased lipogenesis as shown by the higher levels of newly synthetized SFA and MUFA, such as PA and OA (Bartolacci et al., 2017; Singh et al., 2018).

The liaison between oncogenic RAS and lipids seems to consistently occur in cancers other than LC. Indeed, it has been shown that oncogenic KRAS downregulates hormone-sensitive lipase (HSL) in pancreatic cancer, modulating invasion and metastasis (Rozeveld et al., 2020). Pancreatic cancer cells accumulate fat into lipid droplets, which is then used to fuel catabolism during metastasis and invasion. Indeed, blocking the KRAS–HSL axis lowers lipid storage into lipid droplets, effectively reducing invasive capacity of KRAS-mutant pancreatic cancer (Rozeveld et al., 2020). A positive association between high cholesterol:high-density lipoprotein (chol:HDL) ratio and KRAS mutation has been found also in a subset of metastatic CRC (Tabuso et al., 2020). In addition, in murine models of MYC/KRAS breast cancer, FA metabolism genes are upregulated in tumors treated with neoadjuvant therapy, suggesting that this is feature of therapy resistance and recurrence (Havas et al., 2017).

## Oxidative Stress and Oncogenic RAS: The Redox Paradox

**FIGURE 2:**
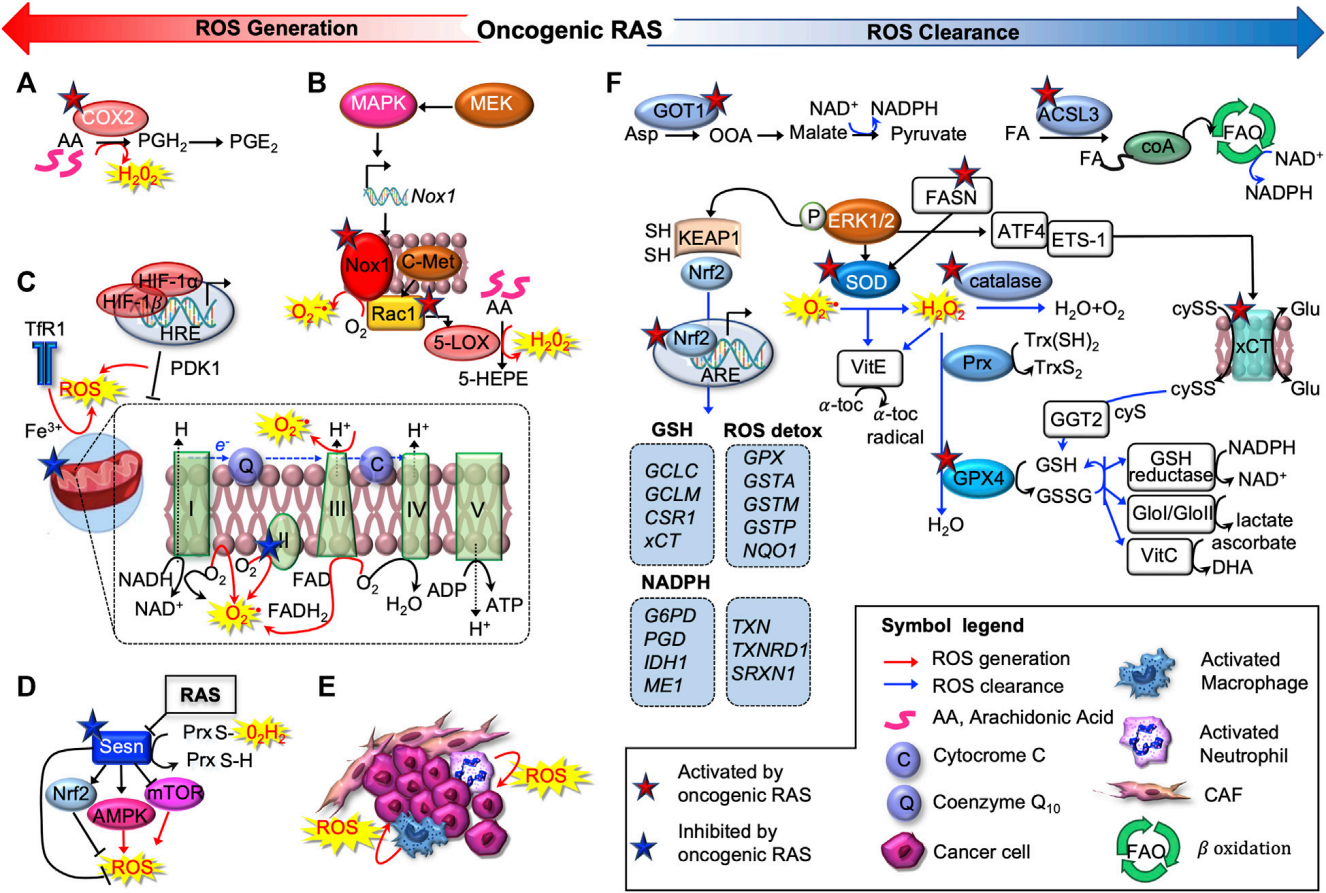
Oncogenic RAS induces pro-oxidant and anti-oxidant programs. Oncogenic RAS promotes ROS production exploiting several strategies, as activation of cyclooxygenase-2 (COX2) **(A)**, subunits of the NADPH oxidase complex (NOX1/4) **(B)**, regulating mitochondrial activity **(C)** or inactivating sestrin 1 (SESN1) **(D)**. The tumor microenvironment (TME) can also produce ROS, contributing to maintain oxidative, pro-tumorigenic conditions **(E)**. Oncogenic RAS drives multiple antioxidant programs as well **(F)**. First, it can upregulate the main antioxidant enzymes superoxide dismutases (SOD); catalase and peroxidases, of which GPX4 is the main member. Oncogenic RAS can drive NADPH production through an alternative glutamine metabolic pathway mediated by aspartate aminotransferase (GOT1), or potentially via a fatty acid oxidation pathway mediated by acyl-coenzyme A (CoA) synthetase long-chain family member 3 (ACSL3). In addition, oncogenic RAS upregulates several key antioxidant proteins, including the light-chain subunit of the system xc−transporter (xCT), nuclear factor, erythroid 2-like 2 (NRF2), and gamma-glutamyltransferase 2 (GGT2).

Cancer cell metabolism and redox signaling are intimately coupled and mutually regulated (Holmström and Finkel, 2014; Wang et al., 2019a): on the one hand, ROS accumulate as by-products of cellular metabolism, on the other, increased ROS and lactate quantities enhance metabolic rate and act as mitogenic signaling molecules, sustaining tumorigenesis (Lee et al., 1999; Ogrunc et al., 2014). However, excessive ROS can cause oxidative damage to macromolecules (*e.g*. DNA and lipids) and can alter intracellular signal transduction (*e.g.* through NF-κB). This is especially true in RAS-driven tumorigenesis: if oncogenic RAS induces ROS accumulation, then ROS scavenging mechanisms must be put in place to reduce cellular senescence and support tumorigenesis (Lee et al., 1999) (Figure 2).

In mutant-RAS cancer cells, high ROS levels can result from increased metabolic activity of peroxisomes, oxidases, cyclooxygenases (COX), lipoxygenases (LOX), from mitochondrial dysfunction, or they can derive from the cross-talk with infiltrating immune cells and other components of the tumor microenvironment (TME) (Szatrowski and Nathan, 1991; Babior, 1999; Storz, 2005).

Oncogenic RAS promotes the direct activation or induction of ROS-producing enzymes. For instance, in murine peripheral lung epithelial cells, mutant KRAS^G12V^ increases levels of intracellular ROS through COX2, which produces hydrogen peroxide (H_2_O_2_) as a by-product of prostaglandin-E2 synthesis (Maciag et al., 2004) (Figure 2A). Several investigations determined that oncogenic RAS increases protein level and activity of NADPH oxidase (NOX), the enzyme responsible for the catalytic one-electron transfer of oxygen at the cell membrane to generate superoxide anion (O_2_^−•^) (Kong et al., 2013; Ogrunc et al., 2014) (Figure 2B). In particular, RAS-driven induction of NOX1 and RAC1 was found to be mediated by the MAPK pathway (Mitsushita et al., 2004). Accordingly, *Nox1* abrogation hampers O_2_^−•^ generation and oncogenic RAS-driven tumorigenesis, NIH3T3 fibroblasts ectopically expressing HRAS^G12V^ have higher amounts of O_2_^−•^ in a Rac1-dependent way as they progress through the cell cycle (Irani et al., 1997). Consistently, in PanIN1b)/PanIN2 stage of pancreatic carcinogenesis, concomitant deletion of tumor protein p53-induced nuclear protein 1 (TP53INP1) and activation KRAS^G12D^, activate Rac1, accelerate PanIN formation and increase pancreatic injury (Al Saati et al., 2013). Active Rac1 was further implicated to induce 5-Lipoxigenase (5-LOX)-mediated generation of H_2_O_2_ and c-Met-triggered O_2_^−•^ production (Shin et al., 1999; Ferraro et al., 2006) (Figure 2B).

In addition, oncogenic RAS was reported to modulate mitochondrial metabolism, hence ROS generation, suppressing the respiratory chain complex I and III (Weinberg et al., 2010; Hu et al., 2012; Liou et al., 2016), regulating hypoxia-inducible factors (HIFs), HIF-1α and HIF-2α (Chun et al., 2010), or the transferrin receptor (TfR1) (Jeong et al., 2016) in CRC and PDA (Figure 2C).

Induction of growth factor- and cytokine-signaling, autophagy-specific genes 5 and 7 (*ATG5, ATG7*) (Kim et al., 2011) or expression of micro RNAs such as miR-155 (Wang et al., 2015) are other ROS-producing mechanisms exploited by RAS. Interestingly, RAS can attain and sustain a prooxidant environment also repressing sestrins (SESN1, 2, and 3), which mediate the regeneration of cytosolic peroxiredoxins (PRXDs), the enzymatic antioxidants involved in the decomposition of endogenously produced H_2_O_2_ (Figure 2D). In MDAH041 immortalized fibroblasts, expression of activated RAS (HRAS^G12V^ and NRAS^G13D^) transcriptionally repressed *SESN* family genes, thus increasing intracellular ROS production (Lee et al., 1999; Kopnin et al., 2007; Zamkova et al., 2013). Finally, many cancers arise from sites of chronic irritation, infection or inflammation. Apart from cancer cells, also various tumor-associated cell types (*e.g.* activated macrophages and neutrophils) produce ROS contributing to maintain an oxidative, pro-tumorigenic TME (Marumo et al., 1997; Basuroy et al., 2009; Edderkaoui et al., 2011) (Figure 2E).

On the other hand, detoxification from ROS can be achieved by the complex battery of antioxidant systems shown in Figure 2F, including both antioxidant enzymes, which specifically scavenge different kinds of ROS, and non-enzymatic molecules, i.e. GSH, flavonoids, and vitamins A (ascorbic acid), C (ascorbic acid) and E (α-tocopherol). RAS-transformed cells upregulate all the three major types of primary intracellular antioxidant enzymes found in mammalian cells: superoxide dismutases (SOD), catalase and peroxidases.

KRAS stably expressed in NIH 3T3 cells, or transiently transfected in COS7 cells, was found to stimulate the scavenging of ROS by posttranscriptionally activating manganese (Mn)SOD, via an ERK1/2-dependent pathway (Santillo et al., 2001). Similarly, HRAS–transduced human keratinocyte HaCaT cells have higher SOD than control cells (Yang et al., 1999). Numerous proteomic analyses performed after RAS-mediated transformation revealed changes in other proteins involved either directly in metabolizing ROS or in maintaining the redox balance, such as Peroxiredoxin 3 and 4, thioredoxin peroxidases, NADH dehydrogenase ubiquinone Fe/S protein, glyoxyalase I, selenophosphate synthetase, and gamma-glutamyltransferase 2 (GGT2) (Young et al., 2004; Recktenwald et al., 2008; Moon et al., 2012). Increased expression of these enzymes was paralleled by an elevated tolerance of KRAS mutants against the cytotoxic potential of H_2_O_2_ and formaldehyde.

Mechanistically, oncogenic RAS activates expression of antioxidant genes predominantly trough the nuclear factor, erythroid derived 2, like 2 (NFE2L2, also known as NRF2), which is widely regarded as the master regulator of antioxidant response (Figure 2F). NRF2 binds to the antioxidant response elements (ARE) within promoters of genes encoding antioxidant enzymes, such as glutathione S-transferase A2 (GSTA2) and NADPH quinone oxidoreductase 1 (NQO1) (Nguyen et al., 2009). For example, KRAS^G12D^ raised mRNA and protein levels of *Nrf2* and its target genes, *e.g*. *Nqo1*, and decreased immunoreactivity for 7,8-dihydro-8-oxo-2′-deoxyguanosine (8-oxo-dGuo), one of the major products of DNA oxidation *in vitro* (Denicola et al., 2011). Importantly, such activation was validated *in vivo*, when comparing KRAS^G12D/+^ pancreatic cancer cells to KRAS^LSL/+^ epithelial cells in murine KRAS PanIN and PDA. Consistently, Nrf2-deficient murine PanIN were negative for Nqo1 and demonstrated similar levels of 8-oxo-dGuo and MDA in PanIN compared to neighboring normal ductal cells (Denicola et al., 2011).

Moreover, NRF2 activity is regulated by a coordinated protein complex consisting of Kelch-like ECH-associated protein 1 (KEAP1), CLLIN3 (CUL3), ubiquitin ligase, and other factors (Taguchi et al., 2011) (Figure 2F). Under normal conditions, this complex mediates the protein degradation of NRF2, preventing its translocation to the nucleus. However, oncogenic RAS can induce conformational changes in KEAP1, resulting in the upregulation of NRF2 target gene transcription and the following cytoprotection (Taguchi et al., 2011).

Noteworthy, all the enzymatic antioxidant activities responsible for ROS detoxification consume GSH and ultimately NADPH. Not only GSH directly scavenges hydroxyl radical (HO^•^) and O_2_^−•^, but it acts as cofactor in antioxidant systems, and it regenerates the active forms of vitamin C and E. Once oxidized, glutathione (GSSG) can be converted back to its reduced form by glutathione reductase (GSR). Thus, the GSH/GSSG ratio can be assumed as an index of the redox buffering capacity of the cell. In order to increase intracellular GSH levels, oncogenic KRAS controls *xCT* transcription by downstream activation of ETS-1 which synergizes with Activating Transcription 4 (ATF4) (Lim et al., 2019) (Figure 2F). xCT (encoded by the gene *SLC7A11*) is the subunit of the system xc^–^transporter, responsible for the exchange of intracellular glutamate for extracellular cystine, which, once inside the cell, is rapidly reduced to cysteine, the rate-limiting precursor in the synthesis GSH (Sato et al., 1999).

Given that NADPH is required to reduce GSSG and is thus the predominant source of reducing power, generation and maintenance of intracellular GSH and NADPH pools is crucial for redox homeostasis and potentially for oncogenesis. This can be achieved by rewiring cellular metabolic circuitries, as glutamine and glucose metabolism. In PDA, mutant KRAS was found to upregulate transcriptionally the aspartate transaminase (GOT1) (Son et al., 2013): in this way, GOT1 converts glutamine-derived aspartate into oxaloacetate, which fuels malate and then pyruvate synthesis, thus increasing the NADPH/NADP^+^ ratio (Figure 2F). In LC cell lines, as well as in lung tumors, KRAS^G12D^ enhances glucose metabolism providing the metabolites to be channeled into the TCA cycle, increasing NADPH levels and ultimately leading to ROS detoxification (Kerr et al., 2016). Moreover, in human LC cells and in lung tumors, mutant KRAS promotes FA oxidation (Padanad et al., 2016), a process that generates acetyl-CoA, which is metabolized to produce NADPH (Carracedo et al., 2013), especially under conditions of glucose scarcity (Figure 2F). Besides the generation of NADPH as a byproduct of FA oxidation, a direct link between lipid metabolism and oxidative stress was suggested by Yun et al., who showed that *FASN* knockdown decreased *SOD* expression, increased ROS production and sensitivity to H_2_O_2_. This report demonstrates how FASN regulates H_2_O_2_-induced cytotoxicity in CRC SNU-C4 (KRAS^G12C^) human cancer cells (Yun et al., 2017).

## Lipid Peroxides at the Cross Node Between Lipids and Oxidative Stress

At physiological levels, lipid peroxides (LOOH) have beneficial effects: they induce cellular adaptive responses and enhance tolerance against subsequent oxidative stress through upregulation of antioxidant compounds and enzymes (Gaschler and Stockwell, 2017). However, their uncontrolled generation finally results in the initiation and execution of ferroptosis. LOOH production preferentially occurs in cell membranes due to the high solubility of molecular oxygen and it can be carried out either in an enzymatic or non-enzymatic manner. Yet, the two LPO mechanisms share the same substrate: PUFA.

PUFA, as LA, AA, DHA, and EPA are defined as long chain FA with two or more carbon-carbon double bonds. PUFA, as free FA or esterified into the sn-2 position of PL, are the preferential substrate of LPO, whereas acyl of the sn-1 position hardly participate in oxidation reactions (Davies and Guo, 2014). Other unsaturated lipids, such as cholesterol, can be oxidized to hydroperoxides too, but to a minor extent (Smith, 1987). Indeed, the bis-allylic hydrogen with a (1Z, 4Z) pentadiene moiety makes the C-H bond in PUFA weaker and the hydrogen more susceptible to abstraction (Gaschler and Stockwell, 2017). As elegantly shown by Yang *et al.*, replacing natural PUFA with deuterated PUFA (dPUFA) which have deuterium in place of the bis-allylic hydrogens, reduced oxidative stress and prevented cell death induced by Erastin or RSL3 -two potent ferroptosis inducers-in HT-1080 fibrosarcoma cancer cells (which harbor NRAS^Q61A^) (Yang et al., 2016). Further, direct evidence for the oxidation of PUFA during ferroptosis was provided by incubating HT-1080 cells with alkyne-labeled LA, followed by copper-catalyzed cycloaddition (Click)-labeling reaction. Treatment with Erastin induced the accumulation of oxidative breakdown products of LA, which could be prevented by cotreatment with Ferrostatin-1 (Fer-1), a potent and selective inhibitor of ferroptosis (Skouta et al., 2014). Consistently with this concept, addition of AA or other PUFA was reported to increase ferroptosis sensitivity, possibly due to their increased incorporation into PL (PUFA-PL) (Conrad et al., 2018). Similarly, Fuentes et al., found that n-3 PUFA specifically suppress oncogenic KRAS-driven CRC by 1) incorporating into plasma membrane PL, 2) modifying KRAS nanoscale proteolipid composition, 3) disrupting oncogenic KRAS driven signaling, and finally 4) suppressing KRAS-associated phenotypes *in vitro* and *in vivo* (Fuentes et al., 2018).

On the contrary, MUFA do not have bis-allylic positions, hence are not readily oxidized. Rather, they can act as potent suppressors of ferroptosis in cancer cells. For instance, Magtanong *et al.* found that exogenous OA and palmitoleic acid (POA; C16:1), upon ACSL3-mediated activation, protected HT-1080 and A549 (NSCL, KRAS^G12S^) cancer cells from ferroptosis induced by Erastin or its more potent analog, Erastin2 (Magtanong et al., 2019; Tesfay et al., 2019).

Interestingly, in regard to the potential impact of dietary FA on cancer, SFA and MUFA, but not PUFA, were associated with increased risk of CRC with specific KRAS mutations at codon 12 (Slattery et al., 2000; Weijenberg et al., 2007). On the contrary, dietary consumption of n-3 PUFA, such as EPA and DHA, results in their incorporation into cell membrane PL (Chapkin et al., 1991) and has been associated with reduced CRC risk (Hall et al., 2008).

The central requirement for PUFA oxidation in ferroptosis is also supported by genetic evidence linking specific lipid metabolic genes to the execution of ferroptosis. In particular, a CRISPR-based genetic screen identified ACSL4 and Lysophosphatidylcholine acyltransferase 3 (LPCAT3) as promoters of RSL3-and DPI7-induced ferroptosis (Dixon et al., 2015; Moerke et al., 2019).

ACSL4 is essential for both lipid metabolism and ferroptosis (Müller et al., 2017). Of all 6 ACSL isoforms, only ACSL4 has been positively correlated with ferroptosis likely because of its marked preference for PUFA (AA and EA, in particular) (Doll et al., 2017). Indeed, it was recently proven that increased levels of long n-6 PUFA are dependent on enhanced expression of *ACSL4*. Hence, ACSL4 has been proposed as both a biomarker and a regulator of ferroptosis. On the contrary, ACSL3 is known to preferentially activate MUFA, OA in particular, thus protecting plasma membrane PL from oxidation, supporting KRAS LC and metastasizing melanoma cells (Padanad et al., 2016; Magtanong et al., 2019; Ubellacker et al., 2020).

LPCAT3 preferentially mediates the insertion of AA into membrane PL by re-acylating LysoPL, mostly lysophosphatidylcholines (LysoPC) and lysophosphatidylethanolamines (LysoPE) (Eto et al., 2012; Wang and Tontonoz, 2019; Bartolacci et al., 2021). However, LPCAT3 can insert both PUFA- and MUFA-CoA esters (Hu et al., 2017; Bartolacci et al., 2021). Thus, our current understanding is that the requirement for LPCAT3 in ferroptosis might depend on the pool of available FA, the cell-type and the ferroptotic stimulus. For instance, LPCAT3 was reported as necessary to mediate RSL3-induced ferroptosis in HT-1080 and Calu-1 cells (Dixon et al., 2014), while we recently reported that *LPCAT3* knockdown drives mutant KRAS NSCLC human cell lines to ferroptosis Bartolacci et al., 2021).

## Enzymatic and Non-enzymatic Lipid Peroxidation: Two Ways to Oxidize PUFA

**FIGURE 3:**
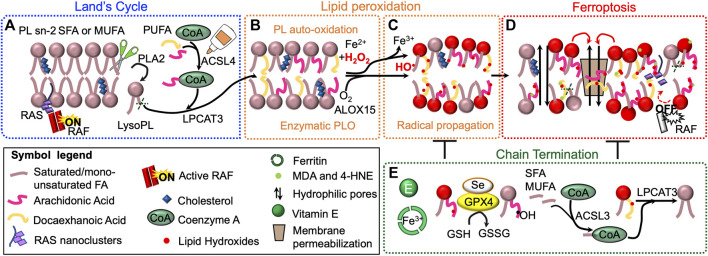
Lipid peroxidation drives ferroptosis. Phospholipid (PL) acyl chain remodeling (Land’s cycle) is responsible for the enrichment of membranes with polyunsaturated fatty acids (PUFA), when monounsaturated (MUFA) and saturated (SFA) FA become limiting. Phospholipase A2 (PLA2) removes acyl chain at sn-2 position. Lysophosphatydilcholine-acyltransferase-3 (LPCAT3) re-esterifies the position using PUFA-CoA, generated by acyl-CoA long-chain family member 4 (ACSL4) **(A)**. Membranes PL enriched with PUFA are prone to undergo iron–dependent lipid peroxidation (LPO) possibly via Fenton chemistry or enzymatic oxygenation (*e.g*. ALOX15) **(B)**. Once produced, lipid hydroxides (LOOH), if not cleared by the cellular antioxidant systems, can propagate LPO to other PUFA-containing PL **(C)**. LPO can lead to ferroptotic cell death (highlighted in red) through several mechanisms **(D)**. First, LOOH can alter membrane properties, which could allow the formation of hydrophilic pores and induce membrane permeabilization (i). Second, lipophilic electrophiles formed during the lipid peroxidation event could affect membrane-bound proteins and their signaling cascade (ii). LOOH can also generate second, more stable and highly reactive LPO products, as malondialdehyde (MDA), and 4-hydroxy-2-nonenal (4-HNE) (iii). Finally, LPO can alter lipidomic signature and affect cancer cell metabolism (iv). Cellular antioxidant systems and phospholipid remodeling can counteract and terminate LPO **(E)**.

Enzymatic peroxidation is mostly mediated by LOX that catalyze the stereospecific insertion of oxygen into PUFA, such as AA and LA (Kuhn et al., 2005, 2015) (Figure 3). Although most LOX prefer free FA as a substrate, some isoforms, including 15-LOX, can directly oxygenate PUFA-PL without prior release of esterified PUFA by phospholipase A2 (PLA2) (Kuhn et al., 1990). Shintoku *et al.* assessed the contribution LOX activity to ferroptosis in oncogenic RAS-expressing cancer cells (Shintoku et al., 2017). They showed that 12/15-LOX inhibitors -such as baicalein and PD146176-as well as siRNA-mediated silencing of *ALOX15* are able to prevent Erastin- and RSL3-induced ferroptosis in HT-1080, Panc-1 (PDA, KRAS^G12D^) and Calu-1 (NSLC, KRAS^G12C^) human cancer cells (Xie et al., 2016). On the contrary, treatment with ALOX15-activating compounds, as (E)-1-(7-benzylidene-3-phenyl-3,3a,4,5,6,7-hexahydroindazol2-yl)-2-(4-methylpiperazin-1-yl) ethenone, accelerated cell death at low doses of Erastin and RSL3 (Shintoku et al., 2017). Besides LOX enzymes, oxidized lipids can also be synthesized in a controlled manner by CYP450 mono-oxygenases and COX (Wang et al., 2019b). Interestingly enough, *PTGS2*, the gene encoding COX2, was the most upregulated gene in BJ-derived cell lines expressing HRAS^G12V^- upon treatment with either Erastin or RSL3 (Yang et al., 2014). Knockdown of *GPX4* also increases *PTGS2* mRNA abundance in this system. However, ferroptotic cell death by Erastin or RSL3 is not affected by using indomethacin, a PTGS-1/PTGS-2 (COX-1/COX-2) inhibitor, suggesting that PTGS2 does not regulate ferroptosis and *PTGS2* upregulation could be rather considered a downstream marker of ferroptosis (Yang et al., 2014). This is consistent with the notion that not all inhibitors of LOX can rescue ferroptosis: rather, the compounds that can inhibit ferroptosis are radical-trapping antioxidants (RTA) that can protect against non-enzymatic peroxidation (Shah et al., 2018). Thus, we can hypothesize that autoxidation rather than the LOX-controlled lipid peroxidation is the final process of ferroptosis.

**FIGURE 4:**
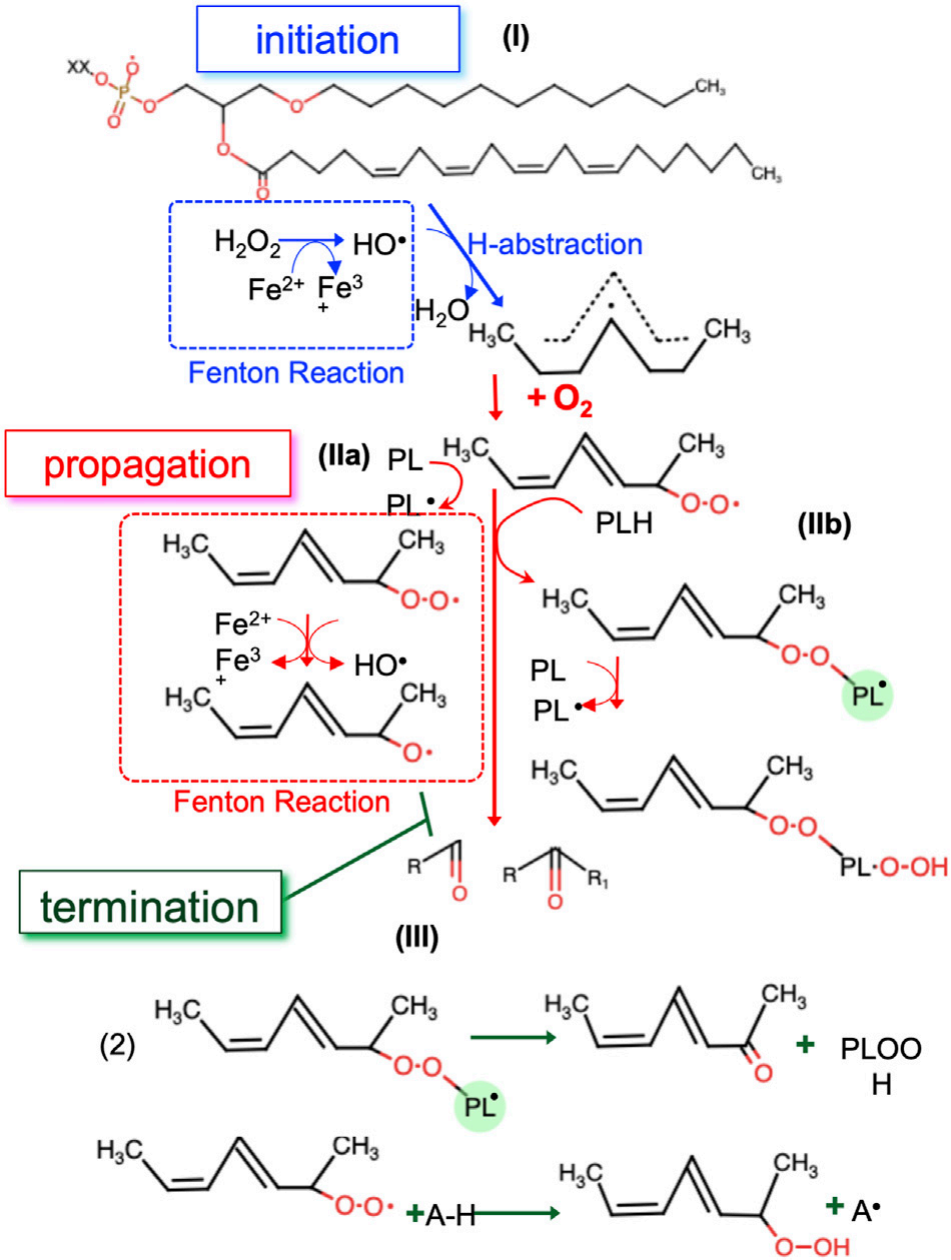
The three steps of non-enzymatic LPO. In the initiation step the first radicals are generated by redox active labile iron **(I)**. In the propagation step radicals are able to react with new substrates, creating new radicals **(II)**. The propagation step repeats until the termination step, where radicals are ‘quenched’ by antioxidants or reacting with another radical **(III)**. •OH, hydroxyl radical; PL, phospholipid; PL-OO^•^, PL peroxyl radical; PL^•^, phospholipid radical, Fe^2+^, ferrous ion, Fe^3+^, ferric ion, PL-O^•^, PL alkoxyl radicals; A-H, antioxidant.

Non-enzymatic LPO can be schematically described in three stages: initiation (I), propagation (II) and termination (III) (Figure 4). Step (I) involves a free radical (i.e., •OH), which abstracts hydrogen from a polyunsaturated acyl chain of a PL. This process can be initiated by any reaction that generates radical compounds from non-radical molecules, often through redox reaction catalyzed by iron. In cells, iron is tightly regulated: it is found mostly ligated by heme, bound in FeS clusters, or to the iron storage protein ferritin (Lane et al., 2015). However, there are small pools of metabolically available, “labile” iron which is loosely ligated, thus able to react with endogenously produced H_2_O_2_ or O_2_^−•^ to form oxygen centered radicals, through a process known as “Fenton chemistry” (Breuer et al., 2008). Interestingly, long-treatment with iron (as ferric ammonium citrate, FAC) strikingly reduced the growth of ovarian carcinoma cells, upon overexpression of HRAS or KRAS (Bauckman et al., 2013). Once formed, oxygen centered radicals readily react with molecular oxygen to form a PL peroxyl radical (PL-OO^•^) (II) (Maillard et al., 1983), which in turn can propagate the reaction in multiple ways. PL-OO^•^ abstracts hydrogen from another PL molecule (Figure 4, **IIa**) and forms PL-OOH and a PL^•^ radical which propagates the chain reaction. In the presence of Fe^2+^, PL-OOH can be converted to PL alkoxyl radicals (PL-O^•^) which also contributes to chain propagation (Buettner, 1993). Alternatively, PL-OO^•^ reacts via addition to the polyunsaturated acyl chain of another PL (Figure 4, **IIb**), which effectively forms PL dimers that are linked via a peroxide bond (Morita and Fujimaki, 1973). These dimers along with other intermediates (PL-OO^•^ and PL-OOH) are instable molecules that suffer decomposition reactions, producing the electrophilic end products of PL autoxidation (reactive aldehydes and oxygenated PL). The free radical chain reaction propagates until two free radicals conjugate to each other to form stable molecules or in the presence of a chain-breaking anti-oxidant (Pratt et al., 2011) (Figure 4, **III**).

## Toxicity of Lipid Peroxides in Ferroptosis-Sensitive Cancer Cells

Once generated, PLOOH, and more in general LOOH, can navigate cells to ferroptosis in several and still not fully elucidated processes (Figure 4D).

### Effects on Membrane

Within the plasma membrane the polar chains in oxidized lipids are energetically unfavorable to stay in the bilayer’s interior. As a result, LPO causes the reversal of the polar lipid chain to the bilayer interface and major changes in membrane properties -*e.g.* increase of area per lipid, bilayer thinning, decreased lipid tail order and increased water permeability (Wong-Ekkabut et al., 2007; Beranova et al., 2010; Cwiklik and Jungwirth, 2010; Boonnoy et al., 2017) (Figure 4D). Moreover, according to atomistic molecular dynamics (MD) simulations, LOOH increase local membrane curvature, hence the accessibility of oxidants into membrane internal leaflet, which if not counterbalanced by GPX4, results in a vicious cycle that will ultimately destabilize the membrane, leading to pores and micellization (Agmon et al., 2018). Consistently, another MD simulation of oxidized lipid bilayers, containing 1-palmitoyl-2-lauroyl-sn-glycero-3-phosphocholine (PLPC) and its aldehyde derivatives, showed that oxidized lipids self-assemble into aggregates with a water pore rapidly developing across the bilayer (Siani et al., 2016). Vitamin E can prevent pore formation by trapping the polar groups of the oxidized lipids at the membrane–water interface resulting in a decreased probability of the oxidized lipids making contact with the two leaflets and initiating pore formation (Wong-Ekkabut et al., 2007). Interestingly, cholesterol and Vitamin E share similar molecular structures (i.e. a hydrophobic tail and a ring structure with a hydroxyl group) that might explain why cholesterol is a less preferred substrate for oxidation, but rather it is associated with increased bilayer thickness, lipid tail order, organized membrane architecture that help circumvent ferroptosis (Saito and Shinoda, 2011; Gilmore et al., 2013). In accordance with these *in silico* findings, when observed by confocal microscopy, Erastin-treated HT-1080 cells stained with LOOH-sensitive probe BODIPY-C11 581/591, show a distinct “ring” of LPO around the plasma membrane and a blister-like deformation with positive curvature (Tarangelo et al., 2018; Magtanong et al., 2019). Importantly, these data reconcile with RAS nanoclustering in cholesterol-poor domains (Zhou et al., 2017) and further indicate the importance of cell membrane composition in dictating ferroptosis sensitivity.

### Effects on Membrane-Bound Proteins

LOOH affect RAS nanoclusters, which are the sites of RAS effector recruitment and activation: as shown by single fluorophore video tracking (SFVT) and electron microscopy (EM) studies, the localization of RAS-GTP to nanoclusters is required for the recruitment and activation of its downstream effector c-Raf (Tian et al., 2007; Zhou and Hancock, 2015) (Figure 4D).

### Generation of Secondary LPO Products and Changes in the Lipidome

LOOH might further break down into many electrophilic species such as aldehydes which are more stable than primary LOOH and can therefore diffuse across membranes and crosslink primary amines on proteins, DNA and other nucleophilic molecules (Esterbauer et al., 1991; Marnett, 1999; Gaschler and Stockwell, 2017; Zhang et al., 2019). Among lipid aldehydes, malondialdehyde (MDA), and 4-hydroxy-2-nonenal (4-hydroxy-2,3-trans-nonenal, HNE) are the most investigated secondary products of LPO (Esterbauer et al., 1991; Kaur et al., 1997) (Figure 4D).

In KRAS human prostate cancer cells, 4-HNE significantly potentiates the antitumor effects of the HDAC inhibitor panobinostat (LBH589) (Pettazzoni et al., 2011). Both single agents and, to a greater extent, their combined treatment induced a G2/M cell cycle arrest in treated cells (Pettazzoni et al., 2011). In KRAS human colon adenocarcinoma cells, 4-HNE was found to inhibit cell proliferation through regulation of the MAP kinase (MAPK) pathway and interacting with transforming growth factor beta (TGF-β) (Vizio et al., 2005). Moreover, KRAS human CRC cells treated with isothiocyanates become resistant to benzo [*α*]pyrene or H_2_O_2_-induced cell death upregulating AKR1C1*,* the enzyme responsible for the reduction of 4-HNE (Bonnesen et al., 2001).

Changes in the lipidome of ferroptotic cancer cells have been widely studied in a variety of cancer models, using different ferroptosis inducers and by different analytic methods. However, it remains to be determined whether such changes are consequential to ferroptosis, or rather have a causative role. For instance, in HT-1080 cells, Erastin induced a depletion of PUFA, *e.g*. LA, EPA and DHA, both as free FA and PUFA-PC cells (Skouta et al., 2014), while increasing the level of LysoPC, which in physiologic conditions represent a minor percentage of cellular membrane lipids (ROBERTSON and LANDS, 1964; Yang et al., 2014). However, when ferroptosis was induced in the same *in vitro* system (i.e. HT-1080 cells), via GPX4-inhibition by FINO_2_, it resulted in the accumulation of a wide array of oxidized PL, i.e. phosphatidylethanolamine (PE), PS, phosphatidylinositol (PI), and cardiolipin (CL) (Gaschler et al., 2018).

Moreover, it should be noted that also wild type (wt) RAS cancer cells undergoing ferroptosis show alterations in their lipidomic profile. As an example, in diffuse large B cell lymphoma (DLBCL) cell lines, IKE decreased levels of LysoPC, PC, PE, and TAG mainly containing PUFA (Zhang et al., 2019). The decrease in TAG upon IKE treatment indicates that in this specific context TAG may be the major oxidation target during ferroptosis, suggesting a possible protective role of this lipid class as a buffer against oxidation stress. However, untargeted lipidomics performed on tumor xenografts of mice treated with a single dose of IKE revealed increases in free FA, PL, and DAG, especially enriched in LA and AA (Listenberger et al., 2003).

These diverse and apparently contradictory results suggest that context specific characteristics (cell membrane composition, tissue of origin, nature of the ferroptosis inducing stimuli) may critically influence the lipids involved in the execution of ferroptosis. Thus, this field remains a very active subject of investigation that will undoubtedly benefit from analytical advances in detecting and quantifying the labile lipid species that are involved in ferroptosis.

## Degradation of Lipid Peroxides to Escape Ferroptosis

**FIGURE 5:**
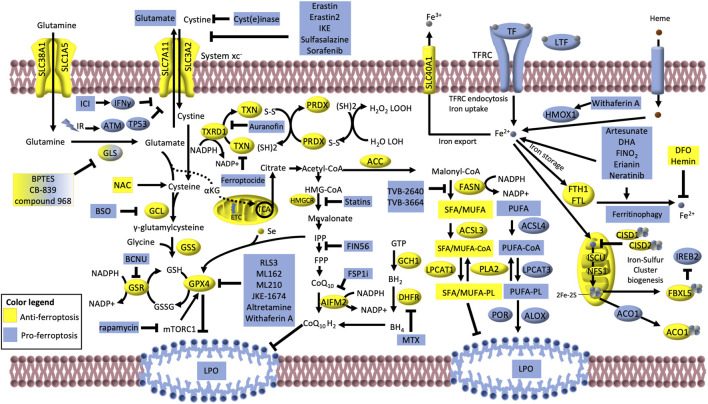
Iron-dependent lipid peroxidation is the hallmark of ferroptosis. The cystine/glutamate transporter, consisting of the SLC3A2 and SLC7A11 (alias xCT) subunits, (collectively known as system xc−) imports cystine in exchange for glutamate. Glutamate is produced via glutaminase (GLS) -dependent glutaminolysis of glutamine. If not exported, glutamate can either be converted into α-ketoglutarate and enter the TCA cycle or participate to glutathione (GSH) synthesis via two sequential reactions catalyzed by glutamate–cysteine ligase (GCL) and glutathione synthetase (GSS). Glutathione peroxidase GPX4 uses GSH to buffer lipid peroxidation (LPO) and protect cells from ferroptosis. The oxidized glutathione (GSSG) is then reduced to GSH via glutathione–disulfide reductase (GSR) using NADPH as electron donor. GSH is a tripeptide antioxidant derived from glutamate, glycine and cysteine, which is turn produced by the reduction of cystine catalyzed by the thioredoxin reductase 1 (TXRD1). Along with the GPX4/GSH system, the TXRD/TXN and the peroxiredoxin (PRDX) systems can convert the phospholipid hydroperoxides (H_2_O_2_ LOOH) to alcohols and water (H_2_O LOH). The AIFM2 (FSP1)–CoQ10 can also counteract LPO and ferroptosis. Moreover, the mevalonate pathway, can indirectly inhibit ferroptosis giving rise to CoQ10 and producing the isopentenyl diphosphate (IPP) that is the precursor for the selenium (Se)-containing GPX4. Also, the GCH1–dihydrofolate reductase (DHFR) system protects lipid membranes from autoxidation catalyzing the biosynthesis of the tetrahydrobiopterin (BH4). Many proteins involved in iron transport, storage and metabolism are key determinants of ferroptosis execution: these include transferrin (TF), lactotranferrin (LTF), transferrin receptor (TFRC), solute carrier family 40 member 1 (SLC40A1), heme oxygenase (HMOX1) and ferritin components (FTH1 and FTL). Also, the mitochondrial proteins cysteine desulfurase (NFS1) and iron–sulfur cluster assembly (ISCU) can reduce the availability of iron by sequestering Fe^2+^ for the biosynthesis of iron–sulfur clusters (2Fe–2S). The iron-regulatory proteins CISD1, CISD2, ACO1 and FBXL5/IREB2 usually negatively regulate ferroptosis. However, under low 2Fe–2S, ACO1 and IREB2 can translationally regulate iron metabolism-related proteins (such as TFRC, SLC11A2, SLC40A1, FTH1 and FTL), thus facilitating ferroptosis. Lipid synthesis and metabolism also play a central role in ferroptosis, by regulating the availability of substrates for LPO. Acetyl- CoA carboxylase (ACC)/FASN axis mediates the synthesis of fatty acids (FA), mainly saturated (SFA) and monounsaturated (MUFA), which have low susceptibility to LPO. SFA/MUFA are conjugated to CoA by the long-chain fatty acid–CoA ligase 3 (ACSL3) prior to be incorporated into membrane phospholipids (PL) via the lysophosphatidylcholine acyltransferase 1 (LPCAT1). On the other hand, Long-chain fatty acid–CoA ligase 4 (ACSL4) and lysophosphatidylcholine acyltransferase 3 (LPCAT3) promote the incorporation of exogenous or lipophagy-derived polyunsaturated FA (PUFA) into PL forming PUFA-PL, which are susceptible to free radical oxidation mediated by lipoxygenases (ALOX) and cytochrome P450 oxidoreductases (POR). Phosholipase A2 (PLA2) can partially counteract this process by cutting out the oxidized FA chains of PUFA-PL. Compounds, proteins, treatments that induce and inhibit ferroptosis are depicted in blue and yellow, respectively, and are discussed in the main text of this review. BSO, buthionine sulfoximine; BCNU, 1,3-bis(2-chloroethyl)-1-nitrosourea; ETC, electron transport chain; GCH1, GTP cyclohydrolase 1; MTX, methotrexate; DFO, deferoxamine; IR, ionizing radiation; ICI, immune checkpoint inhibitor; NAC, N-acetyl cysteine.

To ensure membrane integrity and minimize damages associated with primary or secondary LPO products, cells employ several antioxidant enzymes as described earlier in this review. These defense mechanisms might either detoxify LOOH and/or repair damaged lipids (Girotti, 1998) (Figures 2F, 3E, 5).

Vitamin E acts as a chain breaker to suppress LPO propagation reactions. This might explain why supplementing the diet with the antioxidants vitamin E markedly increases tumor progression and reduces survival in mouse models of KRAS–induced LC (Sayin et al., 2014).

The selenoprotein glutathione peroxidase 4 (GPX4) has been recognized as the master regulator of the enzymatic defense against membrane LPO as it is the only enzyme capable of reducing esterified oxidized FA and cholesterol hydroperoxides (Ursini et al., 1985; Seiler et al., 2008) (Brigelius-Flohé and Maiorino, 2013). Consistently, GPX4 inhibition leads to the rapid accumulation of LOOH, while its overexpression blocks RSL3-induced cell death (Yang et al., 2014; Conrad and Friedmann Angeli, 2015). However, the relation between RAS status and GPX4 is still controversial. For instance, Erastin and RSL3 caused ferroptosis in human tumor cells engineered to express HRAS^G12V^ at lower concentrations than wild-type isogenic cells (Yagoda et al., 2007; Yang et al., 2014; Sui et al., 2018), and inhibiting GPX4 re-sensitized KRAS-expressing NSCLC cell lines (A549 and H460) made radioresistant (Pan et al., 2019). Nevertheless, cancer cells with no oncogenic RAS, as HT29 colon cancer cells, are sensitive to GPX4 inhibition, too (Sui et al., 2018), and ectopic expression of NRAS^12V^, KRAS^12V^, or HRAS^12V^ protects RMS13 rhabdomyosarcoma cells from Erastin-induced apoptosis (Schott et al., 2015).

### *De novo* Lipogenesis

We recently described that mutant KRAS LC deploys *de novo* lipogenesis to limit the amount of PUFA incorporated into membrane PL, deflecting LPO and ferroptosis (Bartolacci et al., 2021) (Figures 3E, 5). These data suggest that mutant KRAS LC leverages lipid synthesis to withstand oxidative stress in the lung environment, which is rich in PUFA and oxygen (Bartolacci et al., 2021). This evidence is consistent with early studies reporting that in hypoxic conditions and in presence of oncogenic RAS, cancer cells scavenge serum lysolipids to meet their needs for SFA and MUFA (Kamphorst et al., 2013), and it provides further mechanistic insights into this dependency.

## Ferroptosis and Oncogenic RAS: A Complicated Relationship

On account of the highly intricate interplay with LPO and oxidative stress, the relationship between oncogenic RAS and ferroptosis is still controversial. On the one hand, pioneer studies in this field reported that expression of oncogenic RAS and/or activation of the RAS/MAPK pathway sensitize cells to ferroptosis inducers (Yagoda et al., 2007; Yang and Stockwell, 2008; Poursaitidis et al., 2017). Additionally, silencing of oncogenic *KRAS* in KRAS-mutant Calu-1 cells significantly reduces the lethality of Erastin. However, the potential link between RAS oncogenes and ferroptosis was later questioned by several observations. Firstly, DLBCL and renal cell carcinoma cell lines, which do not typically contain RAS pathway mutations, outstood as the most sensitive to Erastin sensitivity across a panel of 117 cancer cell lines (Yang et al., 2014). Secondly, RMS13 rhabdomyosarcoma cells ectopically overexpressing oncogenic *HRAS*, *KRAS* or *NRAS* are resistant to Erastin and RSL3 (Schott et al., 2015). However, these findings are in contrast with the observation that EGFR, KRAS, BRAF, and PIK3CA mutations sensitized LC to cystine deprivation–induced death (Poursaitidis et al., 2017). In addition, another study performed on rhabdomyosarcoma and myoblast cell lines showed that cells with high RAS/ERK activation are instead highly proliferative and more susceptible to Erastin and RSL3 (Codenotti et al., 2018).

Reasonable explanations for this apparently confusing picture include the diversity in cell lineage, mutant RAS protein level, proliferative and metabolic status, tumor stage, the existence of niche specific factors and epigenetic changes acquired during tumorigenesis/tumor progression which might contribute to ferroptosis execution/escape.

Many small molecule drugs have been developed to trigger ferroptosis and to inhibit the main enzymes able to metabolize LPO products and/or repair LOOH. Moreover, several FDA-approved drugs that are already in clinical use or have a strong potential for clinical translation were found to promote ferroptosis. Here, we will discuss several therapeutics that are FDA approved or that are being tested in RAS-driven cancers (Figure 5).

### Immunotherapy

Immune checkpoint inhibitors (ICIs) have revolutionized the clinical management of patients with cancer. ICIs act blocking Cytotoxic T-Lymphocyte Antigen 4 (CTLA4), Programmed cell death protein 1 (PD-1) and its ligand PD-L1, thereby activating an effective cytotoxic anti-tumor immune response. Interferon gamma (IFNγ) released from cytotoxic T cells activates the JAK–STAT1 pathway, which in turn downregulates the expression of *SLC7A11* and *SLC3A2* inducing ferroptosis in cancer cells (Wang et al., 2019c) (Figure 5). Moreover, other cytokines released during immunotherapy, such as TGF-ß, can facilitate ferroptosis (Kim et al., 2020). Even though inhibition of PD-L1 failed in KRAS-mutant CRC (Infante et al., 2016), KRAS mutations in NSCLC were predictive of superior response to ICI compared to wild-type patients (Torralvo et al., 2019). Several co-occurring mutations have been described to mediate efficacy of immunotherapy in RAS-mutant LC. Indeed, while TP53 co-mutations are associated with clinical benefit, STK11 (alias LKB1) loss showed ineffectiveness of immunotherapy (Koyama et al., 2016; Dong et al., 2017). It is worth to note that both TP53 and STK11 are involved in ferroptosis regulation. TP53 has been shown to directly or indireclty promote ferroptosis by suppressing SLC7A11 or other metabolic genes (Jiang et al., 2015; Ou et al., 2016; Zhang et al., 2017). On the other hand, LKB1 suppresses ferroptosis via the LBK1-AMPK-ACC-FASN axis (Li et al., 2020a). Therefore, it is tempting to speculate that presence of mutant KRAS and concomitant mutations in TP53 and/or STK11 might influence ICI therapy efficacy by modulating ferroptosis susceptibility.

### Radiotherapy

Radiotherapy is used alone or in combination with other therapies for several solid tumors, including RAS-driven cancers. Radiotherapy has been described to induce ferroptosis in preclinical cancer models and it synergizes with immunotherapy in the suppression of SLC7A11 (Lang et al., 2019) (Figure 5). Ionizing radiation (IR) also activates *ACSL4* expression, thus promoting the formation of PUFA–PL and subsequent LPO (Lei et al., 2020). One more way by which radiation causes ferroptosis is through the release of irradiated tumor cell-released microparticles (RT-MPs) which seem to be at the base of the so-called “radiation-induced bystander effect (RIBE) (Mothersill and Seymour, 2004; Wan et al., 2020). Finally, radiotherapy can promote autophagy-dependent ferroptosis, via activation of cyclic GMP–AMP synthase (cGAS) (Li et al., 2020b). The fact that RAS oncogene has been implicated in establishing radioresistance (Sklar, 1988; McKenna et al., 2003; Wang et al., 2018; Dong et al., 2021) provides the rationale for searching common ground with RAS-induced resistance to ferroptosis in certain cancers (Schott et al., 2015).

### Sorafenib

Sorafenib is an inhibitor of RAF kinases which has being evaluated in clinical trials for several malignancies (NCT03247088, NCT02559778, and NCT00064350). RAF kinases are integral part of the RAS/RAF/MEK/ERK pathway. Therefore cancers driven by RAS have been shown as good candidates for sorafenib treatment (Samalin et al., 2016; Lim et al., 2018; Khoury et al., 2020). Even though sorafenib was reported to induce apoptosis and autophagy in cancer cells trough suppression of RAS/RAF signaling pathway (Ullén et al., 2010; Garten et al., 2019), many other studies suggested that sorafenib induces ferroptosis by inhibiting the system xCT independently of the inhibition of RAF pathways (Dixon et al., 2014; Lachaier et al., 2014; Sun et al., 2016) (Figure 5). Therefore, it is likely that the sensitivity of RAS-driven cancers to sorafenib is due to the susceptibility to ferroptosis induction rather than solely to inhibition of RAS/RAF/MEK/ERK pathway. Future studies and combination trials with other ferroptosis inducers might be useful to understand to which extend ferroptosis contributes to the anticancer effect of sorafenib.

### Sulfasalazine

Sulfasalazine is an anti-inflammatory drug that can suppress the cancer growth by inhibiting the system xCT, inducing ferroptosis in preclinical models (Gout et al., 2001; Dixon et al., 2012) (Figure 5). Sulfasalazine has been evaluated in phase I clinical trials for glioblastoma and breast cancer (NCT04205357, NCT01577966, and NCT03847311). As it regards LC, sulfasalazine has been recently reported to selectively kill KRAS-mutant LC, indicating that it might be a good drug candidate in this tumor type (Hu et al., 2020).

### Cyst(e)inase

Cyst(e)inase is an engineered human enzyme that can degrade cysteine and cystine (cyst(e)ine), causing cell death in cancer cells (Cramer et al., 2017). In particular, cyst(e)inase-mediated depletion of cyst(e)ine is well tolerated and can induce ferroptosis in preclinical models of mutant Kras/Tp53 PDAC (Badgley et al., 2020). These data suggest that strategies regulating extracellular cyst(e)ine levels using cyst(e)inase or cyst(e)ine-deprived diet could offer new therapeutic opportunities in combination with other ferroptosis inducing drugs.

### The Glutamine Metabolism Dilemma

The need of cancer cells for glutamine, the so called “glutamine addiction”, represents a vulnerability that can be exploited therapeutically, especially in KRAS-driven cancers (Son et al., 2013; Toda et al., 2017; Bernfeld and Foster, 2019; Galan-Cobo et al., 2019). Moreover, glutamine, like cysteine, is intimately connected to ferroptosis. If on the one hand, generation of glutamate via GLS1/2-mediated glutaminolysis of glutamine promotes the activity of the xCT system and the synthesis of GSH, on the other hand glutamine is essential to execute ferroptosis under cysteine deprivation (Gao et al., 2015).

Moreover, glutamine contributes to maintenance of the redox balance via the production of aspartate through the transamination pathway. This leads to the formation of malate and pyruvate, concomitantly producing NAD+ and NADPH.

In addition, Muir *et al.* showed that cystine levels dictate glutamine dependence via xCT and concurrent high expression of GLS and xCT may predict response to glutaminase inhibition (Muir et al., 2017). It is unclear whether glutaminase inhibitors like BPTES, CB-839 and compound 968, exert their anticancer effects by modulating ferroptosis sensitivity in KRAS tumor cells and how glutamine dependency might be a predictive marker of ferroptosis susceptibility.

### Neratinib

The tyrosine kinase inhibitor neratinib induces ferroptosis in RAS-, EGFR-, and HER2-driven cancer cells (Booth et al., 2019; Dent et al., 2019, 2020; Nagpal et al., 2019). Neratinib is being tested in trial combination therapy with valproate for advanced RAS-mutated solid tumors (NCT03919292). A further connection between RAS and neratinib is given by recent data showing that RAS-dependent reactivation of mTORC1 accounts for the resistance to neratinib (Sudhan et al., 2020). Therefore, it would be of interest to further investigate whether concomitant RAS/mTORC1 inhibition might synergize with neratinib at inducing ferroptosis.

### GPX4 Inhibitors

RSL3 was first identified in a high-throughput screening as a compound that can selectively induce ferroptosis in transformed cells harboring activated HRAS (Yang and Stockwell, 2008). Affinity purification experiments identified GPX4 as a direct target of RSL3 (Yang et al., 2014) (Figure 5). Similar to RSL3, ML162, another GPX4 inhibitor, was identified in a drug screening for compounds targeting HRAS (Weïwer et al., 2012). However, poor pharmacokinetic properties and promiscuous binding to targets other than GPX4, have limited the use of RSL3 and ML162 in *in vivo* studies and clinical trials (Eaton et al., 2020). On the other end, the pro-drug GPX4 inhibitor ML210 and its derivative, JKE-1674, have shown higher specificity and favorable bioavailability that maybe exploited for cancer therapy (Eaton et al., 2020). Altretamine, an FDA-approved alkylating agent, has been shown to induce ferroptosis (Woo et al., 2015) and was tested in HIV-related lymphoma and sarcoma (NCT00002936). Also the natural compound Withaferin A, has shown a multifaceted pro-ferroptotic activity via inhibition of GPX4, activation of XMOX1, induction of ROS and inhibition of the MAPK/RAS/RAF pathway (Hassannia et al., 2018, 2020; Yin et al., 2020). This pleiotropic effect, targeting multiple dependencies and vulnerabilities of RAS-driven cancers, along with its development into nanocarriers (Hassannia et al., 2018) warrant future investigation to establish whether Withaferin A might be an effective ferroptosis inducer.

### Statins and FASN Inhibitors

Statins are widely prescribed cholesterol-lowering drugs that inhibit HMG-CoA reductase (HMGCR), the rate-limiting enzyme of the mevalonate metabolic pathway, which gives rise to cholesterol (Figure 5). Also, statins block the formation of isopentenyl pyrophosphate (IPP), the precursor of GPX4 and coenzyme Q10, facilitating ferroptosis. Since the mevalonate pathway influences several aspects of the signaling pathways in cancer (Mullen et al., 2016), their potential application in cancer therapy (reviewed in (Longo et al., 2020) has been tested in several tumors, including in RAS-driven cancers. The initial observation that RAS activation may enhance sensitivity to statins (Yu et al., 2018), was then challenged by the failure of several clinical trials (Hong et al., 2014; Lee et al., 2014; Baas et al., 2015). A possible explanation of these outcomes might be that statins induce a feedback activation of the Sterol Regulatory Element-binding transcription Factor 1/2 (SREBP1/SREBP2) pathways which activate the genes of the mevalonate and lipid synthesis. Indeed, suppression of SREBP2 has been reported to sensitize cancer cells to statin-induced death (Longo et al., 2019). Interestingly, mutant KRAS activates the SREBP1/FASN pathway in LC (Gouw et al., 2017) and FASN inhibition is a selective vulnerability of mutant KRAS LC (Bartolacci et al., 2017, 2021). Indeed, the FASN inhibitor TVB-3664 has been reported to induce ferroptosis specifically in KRAS-mutant LC models and its human specific isomer, TVB-2640 is being tested in phase 2 clinical trial KRAS-mutant LC patients (NCT03808558, (Bartolacci et al., 2021). Therefore, we can speculate that combination of statins and SREBP/FASN inhibition might be an efficient strategy to induce ferroptosis in this cancer type.

### Auranofin and Ferroptocide

A combination of the anti-rheumatoid arthritis drug Auranofin and rapamycin is now in phase I, II clinical trial for RAS-mutant small and squamous LC (NCT01737502). Both compounds are being reported to induce ferroptosis and to synergize. Indeed, auranofin induces ferroptosis through inhibition of thioredoxin reductase (TXNRD) activity (Yang et al., 2020) (Figure 5) and has been shown as a successful strategy to induce ferroptosis in small cell lung cancer (SCLC) in combination with BSO-dependent GPX4 inhibition (Bebber et al., 2021). On the other hand, rapamycin, the most used and characterized mTOR inhibitor and inducer of autophagy, has been recently described to induce degradation of GPX4 (Figure 5), thereby activating autophagy-dependent ferroptosis in PDA cell lines (Liu et al., 2021).

Ferroptocide is another molecule targeting the TXN/TXRD system, which induces ferroptosis covalently binding to TXN (Llabani et al., 2019). Of note, TXN is dysregulated in pancreatic cancer where it regulates KRAS signaling pathway (Schultz et al., 2017), indicating that it may represent a good strategy to induce ferroptosis in RAS-driven cancers.

### Methotrexate

Methotrexate is an inhibitor of the dihydrofolate reductase (DHFR), which catalyzes the biosynthesis of the tetrahydrobiopterin (BH4) (Figure 5). BH4 is not only the precursor of nucleotides, but it is also a potent antioxidant that protects lipid membranes from autoxidation. Blocking BH4 synthesis, genetically or via methotrexate treatment, synergizes with GPX4 inhibition at inducing ferroptosis (Soula et al., 2020). Methotrexate is now being tested in combination with regorafenib in phase II clinical trial for recurrent or metastatic KRAS-mutant NSCLC (NCT03520842). Interestingly, methotrexate was initially reported to target RAS by inhibiting the isoprenylcysteine carboxyl methyltransferase. This enzyme is responsible for the carboxyl methylation of RAS protein and its inhibition causes RAS mis localization from the membrane impairing downstream signaling and cell proliferation (Winter-Vann et al., 2003). Ongoing clinical trials and future investigations will determine whether the two mechanisms of action contribute to the anticancer activity of methotrexate in RAS-driven cancers.

### Natural Compounds Inducing Ferroptosis

Several naturally occurring compounds are emerging as potential ferroptosis inducers in RAS cancers. Initially discovered as naturally occurring anti-malarial compounds extracted from *Artemisia annua*, artemisinins have shown potential as anti-cancer therapies (Kiani et al., 2020). In particular, artesunate, one of the most popular artemisinins, can trigger ferroptosis in KRAS-mutant PDA cancer cells by increasing the intracellular levels of free iron (Eling et al., 2015; Wang et al., 2019d). Another natural product, Erianin, isolated from *Dendrobium chrysotoxum Lindl*, has been shown to induce ferroptosis in preclinical models of KRAS-mutant LC by causing high levels of intracellular iron and calcium (Chen et al., 2020). Also, bromelain, a mixture of proteolytic enzymes derived from pineapple stem (*Ananas comosus* L., family Bromeliaceae), has been shown to mediate ferroptosis in KRAS-mutant CRC via upregulation of *ACSL4* (Park et al., 2018).

## Conclusions: The Path Forward

Recent years have witnessed dramatic advancements in our understanding of how cancers driven by oncogenic RAS have altered metabolic needs, leading to the recognition that lipids have roles that go far beyond being simple substrates for energy storage and production. Instead, lipids regulate critical cellular processes. For instance, LPO is involved in the regulation of ferroptosis, a special type of cell death, with potential applications in cancer therapy. In our review of the literature, we explored ferroptosis in the context of oncogenic RAS-driven cancers.

The basic knowledge that has accumulated so far provides an opportunity to reconsider the importance of lipid metabolism and oxidative stress in RAS-driven cancers. However, there is still much work to be done to fully understand RAS metabolic dependencies and their implications in terms of ferroptosis susceptibility. Firstly, it is likely that *RAS* mutations have tissue-specific effects on metabolism. This is due to the intrinsic metabolic wiring in the tissue of origin of a particular tumor and its interaction with oncogenic RAS. In addition, cancer cells undergo a profound lipid metabolism reprogramming during metastasis which in turn may influence their susceptibility to ferroptosis. (Rozeveld et al., 2020; Ubellacker et al., 2020; Ferraro et al., 2021). Also, some evidences have suggested that high proliferative cancer cells are more prone to ferroptosis induction (Codenotti et al., 2018). However, whether the tumor stage and the proliferation rate of *RAS*-driven cancers might affect their susceptibility to oxidative stress and ferroptosis, remains to be elucidated. These and other cancer specific features may create distinct metabolic dependencies for *RAS* mutations in different tumor types that should be explored in a systematic fashion. In a similar manner, *RAS* mutations act in the context of co-occurring mutations—namely other oncogenic events as well as deletion/mutation of a constellation of tumor suppressor genes. For instance, the tumor suppressor p53 has been shown to have an impact on multiple facets of lipid metabolism and ferroptosis (reviewed by (Liu et al., 2020)). Therefore, it will be important to consider the tumor suppressor background when studying the interplay among mutant RAS/lipid metabolism/ferroptosis. Another aspect that requires additional study will be how these RAS-dependent metabolic changes are altered *in vivo* in the TME. This includes areas of hypoxia, limited nutrients, as well as potential metabolic crosstalk between tumor and stromal cells. To understand these complex relationships will require the use of sophisticated autochthonous tumor models as well as the ability to perform metabolic tracing studies *in vivo*. Additionally, in regard to therapeutic targeting of altered lipid metabolism and/or ferroptosis inducers, it will be of significance to identify adaptive responses of *RAS*-driven cancers which could promote therapeutic resistance. As new approaches in lipidomics are applied to the study of ferroptosis in RAS-driven cancers, we anticipate that new biomarkers will be identified, the mechanisms behind ferroptosis-susceptibility will unfold and inform how to integrate ferroptosis inducers with existing chemotherapeutic agents.
